# Primary aldosteronism concurrent with subclinical Cushing’s syndrome: a case report and review of the literature

**DOI:** 10.1186/s13256-020-2353-8

**Published:** 2020-02-20

**Authors:** Yingxiao Zhang, Jianyu Tan, Qin Yang, Zhipeng Du, Shumin Yang, Wenwen He, Ying Song, Jinbo Hu, Yi Yang, Qifu Li, Yao Zhang, Yunfeng He, Qingfeng Cheng, the Chongqing Primary Aldosteronism Study (CONPASS) Group

**Affiliations:** 1 Department of Endocrinology, The First Affiliated Hospital of Chongqing Medical University, No.1 Youyi St., Chongqing, 400016 China; 2 Department of Urology, The First Affiliated Hospital of Chongqing Medical University, No.1 Youyi St., Chongqing, 400016 China

**Keywords:** Primary aldosteronism, Subclinical Cushing’s syndrome, Adrenal vein sampling, Subtype

## Abstract

**Background:**

The prevalence of primary aldosteronism concurrent with subclinical Cushing’s syndrome was higher than previously thought. Through analyzing a rare clinical case, we summarized the diagnosis and management of primary aldosteronism with subclinical Cushing’s syndrome.

**Case presentation:**

A 54-year-old Chinese man of Han nationality was diagnosed as having primary aldosteronism with subclinical Cushing’s syndrome. An abdominal computed tomography scan revealed a mass in his left adrenal gland and a mass in his right adrenal gland. After finishing sequential adrenal venous sampling without adrenocorticotropic hormone, the result reminded us that the left and right nodules were responsible for hypercortisolism and aldosterone hypersecretion, respectively. Right and left adrenalectomy were performed successively. The pathological diagnosis was adrenocortical adenoma for both. Histological findings revealed that the right one had positive immunostaining for CYP11B2 and the left one had positive immunostaining for CYP11B1. The immunohistochemistry result helped us to confirm the diagnosis. Somatic *KCNJ5* mutation (Leu168Arg) was found in the right tumor; there was no *KCNJ5* mutation in the left adrenal tumor.

**Conclusions:**

We suggest that patients with primary aldosteronism should have a low-dose overnight dexamethasone suppression test to screen for hypercortisolism. It can help avoid misdiagnoses and contribute to proper understanding of the adrenal vein sampling result. Making sure of the nidus of aldosterone and cortisol secretion is crucial for the therapy of patients with primary aldosteronism and subclinical Cushing’s syndrome.

## Background

Primary aldosteronism (PA) is a relatively common cause of secondary hypertension by excess aldosterone production from aldosterone-producing adenoma or idiopathic hyperaldosteronism in most cases [1]. Subclinical Cushing’s syndrome (SCS) is a disease whose laboratory characteristics accord with Cushing’s syndrome, but the typical clinical manifestation of Cushing’s syndrome is absent [2]. The prevalence of PA concurrent with SCS is higher than previously thought [3]. We present a rare case of bilateral adrenal tumors in which the left adrenocortical tumor produced cortisol and the right adrenocortical tumor secreted aldosterone, and we review literature on PA concurrent with SCS.

## Case presentation

A 54-year-old Chinese man of Han nationality, a retiree, went to a local hospital because of hematuria. He had an abdominal computed tomography (CT) scan showing right renal mass and bilateral adrenal nodules. He was referred to our hospital for further examination and treatment. He denied any medical history, except for a 4-year history of hypertension. He took 5 mg amlodipine besylate every day. He was married and living with his family; he had smoked tobacco for 30 years and denied alcohol consumption. Members of his family had no history of endocrine diseases or malignant tumors.

At the time of admission, his temperature was 37 °C, the pulse was 80 per minute, and his blood pressure was 161/75 mmHg. His height was 168 cm and weight 64 kg. There was no physical sign of Cushing’s syndrome, such as central obesity, skin atrophy, buffalo hump, red striae of skin, or moon face. The results of his cardiovascular, respiratory, abdominal, and neurological examinations were all unremarkable. A laboratory examination (Table 1) showed an electrolyte disturbance, in particular, a very low serum potassium level. His 24-hour urinary free cortisol was elevated. The function of his thyroid gland, parathyroid hormone, metanephrine, and normetanephrine were in normal range.

**Table 1:** Laboratory and endocrinological data

Test	Result
Blood routine examination	
White blood cells	6.46 × 10^9^/L
Red blood cells	5.14 × 10^12^/L
Hemoglobin	152 g/L
Platelets	224 × 10^9^/L
Biochemistry	
Total protein	68 g/L
Albumin	44 g/L
Aspartate aminotransferase	15 U/L
Alanine aminotransferase	11 U/L
Blood urea nitrogen	5 mmol/L
Creatinine	86 umol/L
Na	149 mmol/L
K	1.9 mmol/L
Cl	98 mmol/L
Total cholesterol	3.91 mmol/L
Fasting blood glucose	4.8 mmol/L
Urinary excretion	
Cortisol	2306.06 nmol/24 hours
Function of thyroid gland	
Triiodothyronine	0.93 ng/ml
Thyroxine	6.08 μg/dl
Free triiodothyronine	2.63 pg/ml
Free thyroxine	0.90 ng/dl
Hypersensitive thyroid-stimulating hormone	1.48 uIU/ml
Other hormones	
Parathyroid hormone	52.5 pg/ml
Metanephrine	118.6 ng/L
Normetanephrine	109.2 ng/L

The endocrine findings (Table 2) showed high plasma aldosterone level and low plasma renin concentration. He then underwent a captopril challenge test which did not show suppressed level of plasma aldosterone concentration. A diagnosis of PA was of no doubt. On the other hand, the circadian rhythm of cortisol disappeared, and his serum cortisol level was not suppressed by an overnight dexamethasone suppression test (ODST) and low-dose dexamethasone suppression test (LDDST). He was diagnosed as having SCS.

**Table 2:** Endocrinological examinations

Examinations	Result		
Endocrinology data			
Plasma aldosterone concentration (pg/ml)	454		
Plasma renin concentration (uIU/ml)	0.83		
Captopril loaded test			
Time (minutes)	0	120	
Plasma aldosterone concentration (pg/ml)	443	467	
Plasma renin concentration (uIU/ml)	0.4	0.4	
Circadian rhythm			
Clock time	8:00	12:00	24:00
Adrenocorticotropic hormone (pg/ml)	34.87	13.37	6.95
Cortisol (nmol/L)	524.32	193.58	166.65
Dexamethasone suppression tests			
Dexamethasone	1 mg	2 mg	
Cortisol (nmol/L)	99.48	72.16	

**Fig. 1:**
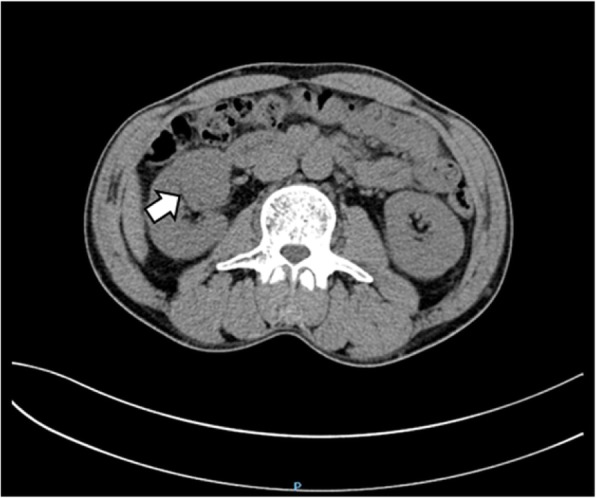
Plain computed tomography scan shows mass in the right kidney. Arrow: kidney tumor

**Fig. 2:**
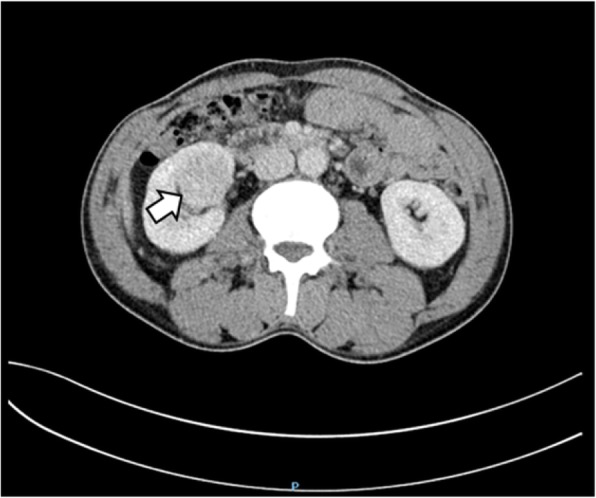
Contrast computed tomography scan shows mass in the right kidney. Arrow: kidney tumor

**Fig. 3:**
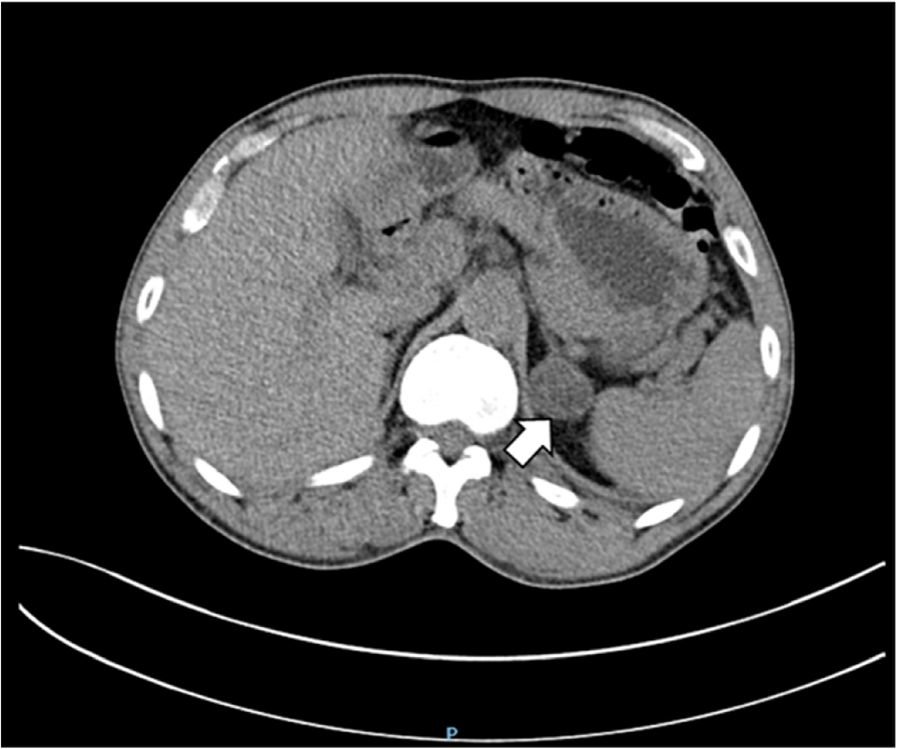
Plain computed tomography scan shows mass in the left adrenal gland. Arrow: left adrenal tumor

**Fig. 4:**
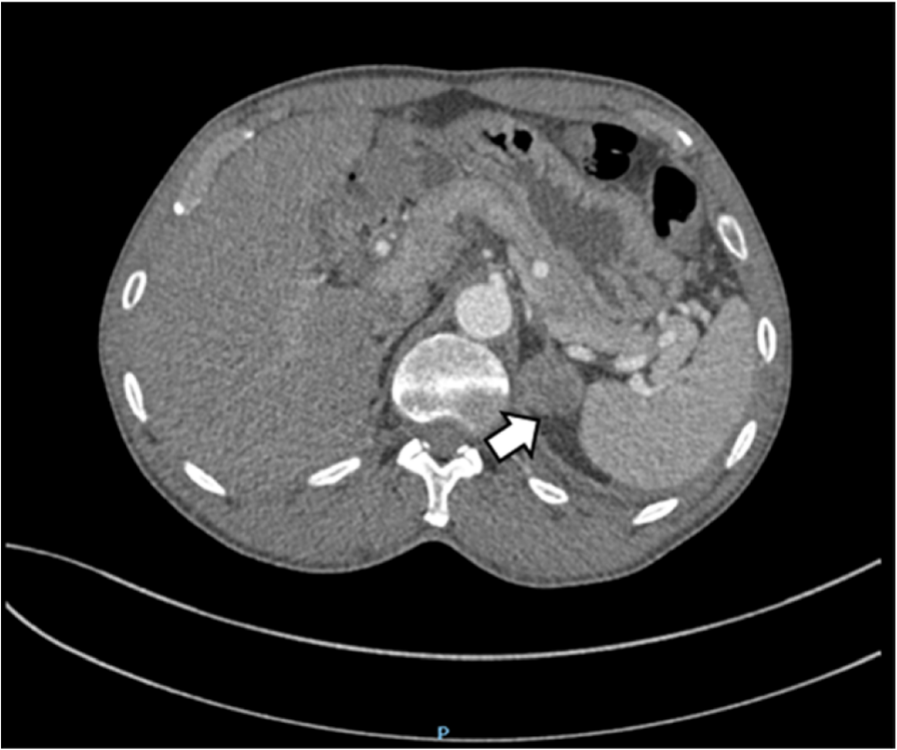
Contrast computed tomography scan shows mass in the left adrenal gland. Arrow: left adrenal tumor

**Fig. 5:**
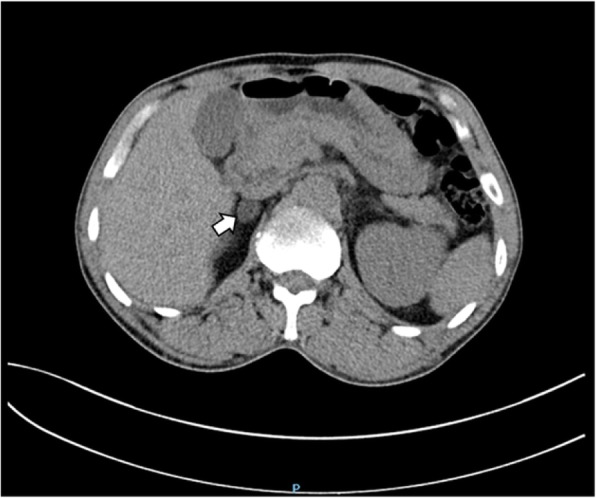
Plain computed tomography scan shows mass in the right adrenal gland. Arrow: right adrenal tumor

**Fig. 6:**
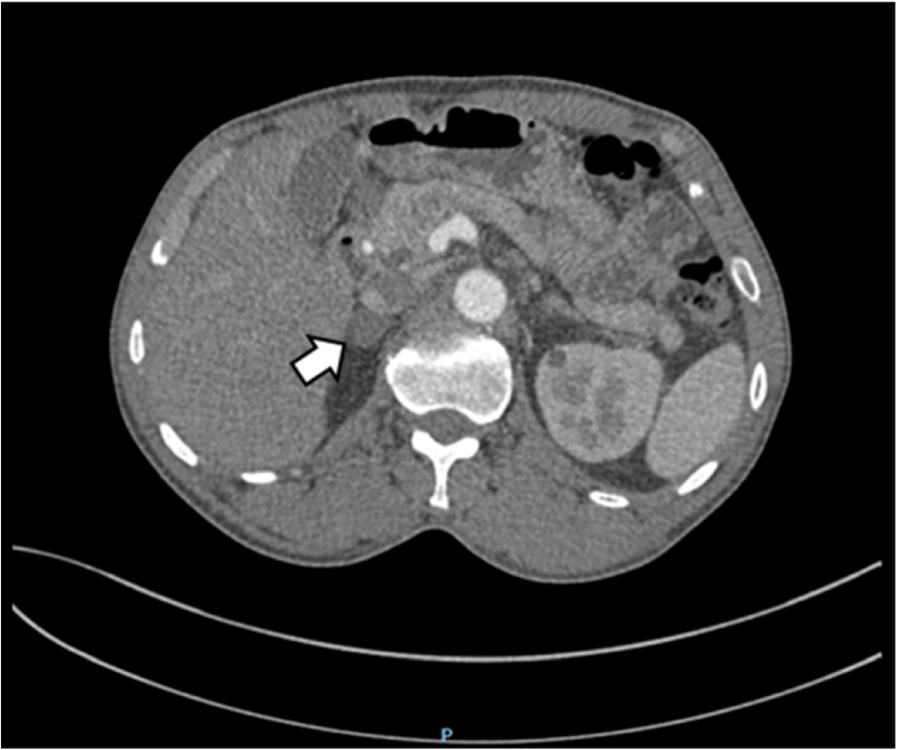
Contrast computed tomography scan shows mass in the right adrenal gland. Arrow: right adrenal tumor

An abdominal CT scan revealed a mass in the parenchyma of his right kidney (Figs. 1 and 2) and there was a rounded, low-density mass in his right adrenal gland and a rounded, low-density mass in his left adrenal gland (Figs. 3, 4, 5, and 6); the larger adrenal mass was approximately 2.6 cm × 2.3 cm on the left adrenal gland. These findings indicated the possibility of a renal tumor and bilateral adrenocortical adenomas.

Sequential adrenal venous sampling (AVS) without adrenocorticotropic hormone (ACTH) stimulation was performed next. The result of AVS (Table 3) reminded us that the right adrenal gland was responsible for aldosterone hypersecretion, and, quite possibly, the left was responsible for hypercortisolism.

**Table 3:** The result of adrenal vein sampling

Sample	Aldosterone (pg/ml)	Cortisol (nmol/L)	Selectivity index	Aldosterone/cortisol ratio
Left adrenal vein	456.00	1268.42	5.63	0.36
Inferior vena cava	420.00	225.10		1.86
Right adrenal vein	10,500.00	294.49	1.78	35.65
Inferior vena cava	336.00	165.54		2.02

Selectivity index: cortisol of adrenal vein/cortisol of inferior vena cava

Right nephrectomy and right partial adrenalectomy were performed first. The golden-yellow nodule in the right adrenal was approximately 2.5 cm × 2 cm × 0.8 cm macroscopically. The pathological diagnosis of the right adrenal nodule was adrenocortical adenoma. The right kidney tumor was proven to be a fibroma. Three days after operation, our patient’s serum potassium was 4.5 mmol/L without any supplementary treatment. The hypertensive drug was changed to 2 mg terazosin every day.

A laparoscopic left partial adrenalectomy was conducted 1 month later. When admitted to hospital, his blood pressure was 127/83 mmHg controlled by 2 mg terazosin every day, and his level of potassium was 4.9 mmol/L without any supplementary treatment. We examined his cortisol at 8 a.m. before operation, which was 344.81 nmol/L. The operation was successful and the level of cortisol at 8 a.m. had reduced to 192.01 nmol/L 2 days after operation. The nodule in the left adrenal was golden-yellow; it was approximately 3 cm × 2.5 cm × 2 cm. The pathological diagnosis of the left adrenal gland nodule was proven to be adrenocortical adenoma. After the second operation, doctors advised 30 mg hydrocortisone should be taken once a day with a weekly reduction of 10 mg until withdrawal, and 2 mg terazosin should be taken once a day.

During postoperative follow-up, terazosin was gradually stopped, and blood pressure and serum potassium remained normal. Approximately 2 months later, his ACTH concentration was 98.73 pg/ml which was above the reference value. This meant our patient had adrenocortical insufficiency, and he continued to take hydrocortisone. Months later, hydrocortisone was stopped, the concentration of cortisol and ACTH were 233.46 nmol/L and 49.43 pg/ml, respectively. The plasma aldosterone concentration was 104 pg/ml and plasma renin concentration was 3.1 uIU/ml. Normalization of aldosterone-to-renin ratio (ARR), cortisol, and ACTH were reached and our patient did not complain of any discomfort.

**Fig. 7:**
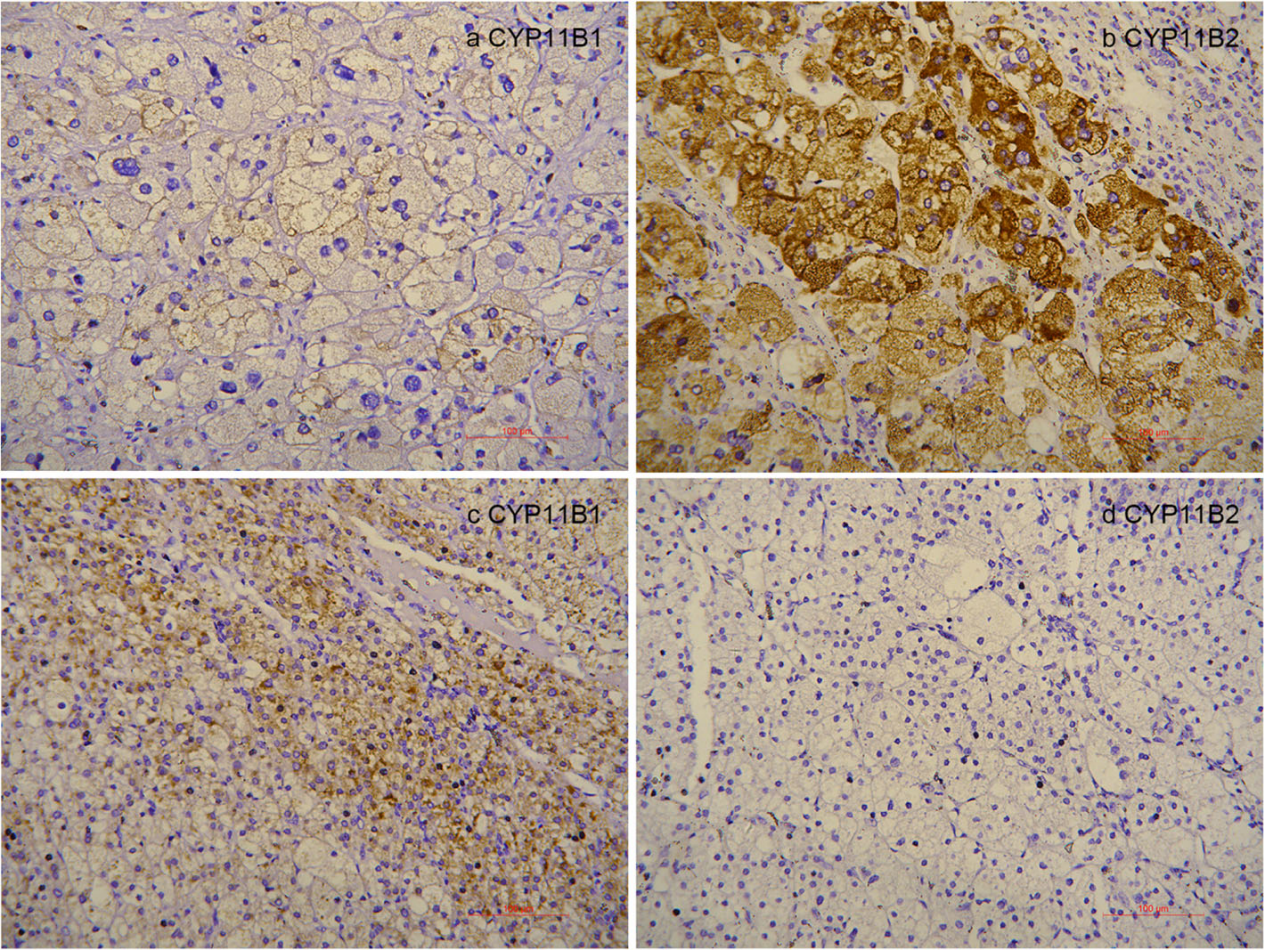
CYP11B1 immunohistochemistry of right adrenal nodule (**a**). CYP11B2 immunohistochemistry of right adrenal nodule (**b**). CYP11B1 immunohistochemistry of left adrenal nodule (**c**). CYP11B2 immunohistochemistry of left adrenal nodule (**d**)

**Fig. 8:**
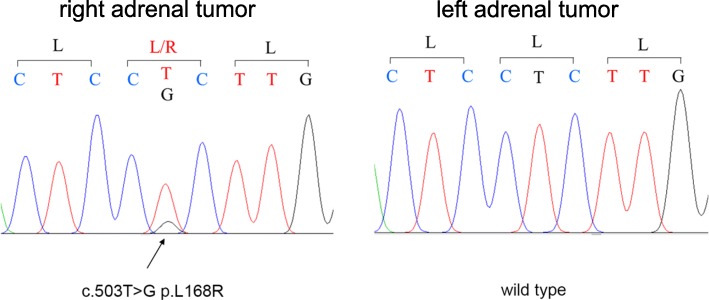
The result of *KCNJ5* DNA sequencing

Immunohistochemistry (Fig. 7) of the two adrenal nodules revealed that the right one had positive immunostaining for CYP11B2, which is essential to synthesize aldosterone, and the left one had positive immunostaining for CYP11B1, which is essential to synthesize cortisol. These results confirmed the right nodule was responsible for the secretion of aldosterone and the left nodule was responsible for the secretion of cortisol. Somatic *KCNJ5* mutation (Leu168Arg) was found in the right adrenal tumor (Fig. 8), and there was no *KCNJ5* mutation in the left adrenal tumor.

## Discussion

This was a rare case of bilateral adrenal tumors, in which the left adrenocortical tumor produced cortisol and the right one secreted aldosterone. The diagnosis was supported by endocrine findings before and after surgery and immunohistochemical evaluation of steroidogenic enzymes. We identified the type of mutation in two adenomas. However, there are some limitations to our study. The usual surgery management of aldosterone-producing adenoma is total adrenalectomy, but a partial adrenalectomy was conducted on our patient because of the fear of adrenal crisis after surgery of both adrenal glands. We did not measure hormones such as metanephrine in the adrenal vein sample, making the interpretation of AVS difficult. And the follow-up visits were not perfect: we did not repeat dexamethasone suppression test and 1 mg ODST after surgery.

Usually, cortisol and aldosterone are secreted by the same adenoma when endocrine findings indicate PA associated with SCS, and CT shows only one nodule in unilateral adrenal gland [4–8]. However, there is not always a single adenoma, so the result of CT can confuse our perception of the nidus of aldosterone or cortisol secretion. Hiraishi *et al.* classified PA associated with SCS by the result of CT, AVS, histology, and immunohistochemistry into six kinds of subtype derived from their eight cases [9]. Although not every case finished all the examinations, such as AVS or histology, some subtypes were speculated; these subtypes still shined a light on our confusion and reminded us that different therapeutic approaches may depend on the disease subtype.

The importance of AVS in the subtype diagnosis of PA is remarkable; AVS should also be performed in patients with PA associated with SCS, which may decide the therapeutic strategy. However, the study of Goupil *et al.* showed that concomitant adrenal autonomous cortisol and aldosterone secretion have potential to confound the lateralization of AVS [10]. The excess production of cortisol may lower the aldosterone/cortisol ratio on the side of the lesion while the opposite side increases due to contralateral cortisol suppression, thereby resulting in an incorrect diagnosis. Goupil *et al.* avoided the problem by measuring the concentration of metanephrine in the venous blood instead of cortisol [10]. They reported a case whose AVS result was bilateral aldosterone production by using aldosterone/cortisol ratio; however, the result of changing to aldosterone/metanephrine ratio showed aldosterone production lateralized to the left with right suppression. The patient underwent surgery and the case was pathologically proved. At last, the patient’s hypertension improved and the PA and hypercortisolism were cured, which was confirmed by negative postoperative fludrocortisone suppression and ODST.

## Conclusion

Due to the high prevalence of hypercortisolism in patients with PA, we suggest performing a low-dose overnight dexamethasone suppression test to screen hypercortisolism before surgery and AVS. If hypercortisolism is identified, hormones such as metanephrine in the adrenal vein sample should be measured. Not only can this prevent misunderstanding of the AVS result, but also help to avoid adrenal crisis after removal of the tumor.

## Data Availability

The datasets analyzed during the current study are available from the corresponding author on reasonable request.

## Abbreviations

ACTH: Adrenocorticotropic hormone
ARR: Aldosterone-to-renin ratio
AVS: Adrenal venous sampling
CT: Computed tomography
LDDST: Low-dose dexamethasone suppression test
ODST: Overnight dexamethasone suppression test
PA: Primary aldosteronism
SCS: Subclinical Cushing’s syndrome
